# Immune Checkpoint Inhibitors in Human Glioma Microenvironment

**DOI:** 10.3389/fimmu.2021.679425

**Published:** 2021-07-09

**Authors:** Amina Ghouzlani, Sarah Kandoussi, Mariam Tall, Konala Priyanka Reddy, Soumaya Rafii, Abdallah Badou

**Affiliations:** ^1^ Cellular and Molecular Pathology Laboratory, Faculty of Medicine and Pharmacy, Hassan II University, Casablanca, Morocco; ^2^ Faculty of Medicine, Medical University of Pleven, Pleven, Bulgaria

**Keywords:** Glioma, immune response, immune checkpoint, immunotherapy, Glioblastoma

## Abstract

Gliomas are the most common primary brain tumors in adults. Despite the fact that they are relatively rare, they cause significant morbidity and mortality. High-grade gliomas or glioblastomas are rapidly progressing tumors with a very poor prognosis. The presence of an intrinsic immune system in the central nervous system is now more accepted. During the last decade, there has been no major progress in glioma therapy. The lack of effective treatment for gliomas can be explained by the strategies that cancer cells use to escape the immune system. This being said, immunotherapy, which involves blockade of immune checkpoint inhibitors, has improved patients’ survival in different cancer types. This novel cancer therapy appears to be one of the most promising approaches. In the present study, we will start with a review of the general concept of immune response within the brain and glioma microenvironment. Then, we will try to decipher the role of various immune checkpoint inhibitors within the glioma microenvironment. Finally, we will discuss some promising therapeutic pathways, including immune checkpoint blockade and the body’s effective anti-glioma immune response.

## Introduction

The immune system is made up of several cell types, which defend the body against possible pathogens (1).

Gliomas or glial tumors are the most common primary brain tumors and they account for 81% of all malignant ones. Although they are relatively rare, gliomas cause significant morbidity and mortality. Glioblastoma is the most aggressive and common (45%) of all 6 glioma types and grades, and presents with a median survival of around 15 months (2).

For more than 10 years of research there has been no significant progress in Glioma treatment until now (3) and the lack of effective treatment for glioma can be explained by the many strategies that cancer cells use to escape the immune system (4).

Immunotherapy is an immunological treatment which uses the host’s immune system to recognize and eliminate cancer cells. Indeed, this type of treatment has been shown to be fairly effective against various types of cancer (3), especially with the blockade of inhibitory immune checkpoint molecules (5). These immune checkpoints control the interactions between T cells and cancer cells through the inhibition or activation of T cells. This process occurs according to the organism’s needs and action of the tumor (3). Moreover, immunotherapy, which acts on immune checkpoint inhibitor’s blockade, has improved patients’ survival in different types of cancers. This new hope of cancer therapy remains one of the most promising approaches for the effective activation of therapeutic antitumor immunity (6).

In recent years, these observations have raised the curiosity of researchers who have taken a great interest in immune checkpoint blockade in glioma. Previous studies have shown that the combination of anti-PD-1 and anti- CTLA-4 blocking Abs, does not improve the overall survival [(7), p. 143].

In addition, no obvious benefit of neoadjuvant nivolumab was obtained with resectable glioblastoma (GBM), and presented with a median overall survival of just 7.3 months (8). Similarly, a phase III trial comparing nivolumab (anti-PD-1 blocking Ab) to bevacizumab (anti-VEGF blocking Ab) on patients with recurrent GBM failed to substantiate the benefit of nivolumab, and conferred a similar median overall survival (mOS, 9.8 vs 10.0 months) (9).

The present review aims at describing the immune response within the glioma microenvironment, and discussing the involvement of various immune checkpoint inhibitor molecules used by glioma cells in order to escape the immune response. It will also report some potential therapeutic pathways which involve immune checkpoints blockade.

## Immune Response Within the Brain

It is becoming more and more accepted that there exists an intrinsic immune system which is present and functional in the central nervous system (CNS) (10). However, in the 20^th^ century, the brain was defined as a privileged organ, which meant that the brain and meninges were devoid of lymphatics (11). It was initially presumed that the physiological characteristics of the CNS, absence of antigen-presenting cells (dendritic cells) and presence of the blood brain barrier (BBB) were the causes of the lack of immune surveillance in the brain. Previous studies have shown that upon infiltration of bacteria and viruses, the immune system response could not be recognized or established (12). Hence, when Lowenstein et al. transplanted skin grafts into the brain of non-immunized animals, they found that it did not elicit an immune response (13). These studies led to the belief that the brain is a privileged organ (11, 13).

However, in October 2015, a study conducted by Louveau et al. on mice showed that the brain, like every other tissue, is connected to the peripheral immune system (10).

Through the use of novel techniques, such as staining mice’s meninges with immunohistochemistry, they were able to highlight that endothelial cells, T cells and MHC II-expressing cells were the most present near the dural sinuses. Upon resection of the deep cervical lymph nodes, there was an accumulation of meningeal T-cells due to an inability of T-cells to drain from the meningeal space (14). Hence, the suggestion that the primary route of drainage is from the meningeal vessels (15). The basis of this new theory was a report by Aspelund et al. where they discovered that the Schlemm’s canal in the eye acted like a lymphatic vessel (16). This led other scientists to hypothesize that similar vessels may also be present within the brain and to question the extent of the brain’s immune privilege (10).

Innate immunity-related molecules like cytokines, toll-like receptors and the major histocompatibility complex are expressed in the brain and they influence the generation of an efficient immune microenvironment (17). These cellular constituents elicit an immune response which further supports the idea that the brain does in fact have immune surveillance. Microglia cells are the most predominant and make up 80% of the immune cells in the brain (18). Others include dendritic cells, B-cells, and T-cells, of which B-cells are the most abundant (15). The movement of immune cells and fluid from the CSF is made possible by the lymphatic system lining the dural sinuses (10). Cytokines are signaling proteins and are mostly secreted by immune cells. They can be described as pro-inflammatory or anti-inflammatory (19). It was originally thought that cytokines could not pass through the BBB *via* membrane diffusion as they were too large and hydrophobic. However, cytokines have overcome this by using saturable transport systems or by passing through the disrupted parts of the BBB (20).

Chemokines are low molecular weight proteins that are involved in direct cell migration. They attract leukocytes to the site of infection to allow the mediation of acute and chronic inflammation (21).

In normal circumstances, however, their expression is diminished. Homeostatic chemokines are involved with maintaining leukocyte composition in preparation for an immune response to an insult. Still, inflammatory chemokines are produced during infections or in response to an inflammatory stimulus (22). The immune response in the brain progresses at a much slower rate as opposed to that in the peripheral tissue (14).

Microglia are the tissue resident macrophages of the brain and are involved in innate immunity and infection. They are the largest source of inflammatory mediators in the brain and are derived from hematopoietic precursor cells of the yolk sac and are defined as CD11b+/CD45 low (23).

Microglia are incredibly important for the regulation of angiogenesis and vascularization, which plays a key role in tumor development. In pathological states, injury serves as an example; there is microglia-mediated neuronal injury and glial cell injury through the production of proinflammatory factors like cytokines and chemokines (20). Following this activation, inflammatory molecules are released, which, in turn, activates astrocytes and cells of the immune system. In this disease state, the activated cytokines and the chemokines are essential in maintaining the immune surveillance (22).

Cancer cells are capable of avoiding recognition and cancer immune-editing can be conceptualized into three phases : Elimination, Equilibrium and Escape (17, 24). However, when the tumor cells escape immune recognition, they progress to a clinical stage of cancer and mark the escape stage of immune-editing. Tumors are able to escape either because of tumor induced immunosuppression or because of immune system deterioration (17).

## Glioma Microenvironment

Primary tumors of the central nervous system account for only 2% of all tumors. Despite their low incidence, they are highly prevalent in small children, adolescents and young adults with relatively high mortality and morbidity (25). Gliomas are the most common primary central nervous system (CNS) tumors (26), and are classified according to grades (I to IV) of the World Health Organization (WHO) (27). Thus, gliomas are divided into two groups according to the malignancy of the tumor: tumors of low grades (grades I and II), which have slow growth, and high-grade gliomas (III and IV), which strongly infiltrate the brain parenchyma (28).

To date, glioblastoma is the most aggressive glioma and the deadliest of all (29). Even with the current treatments, namely surgical resection, radiotherapy, and chemotherapy (30), it is still an incurable disease with a fairly poor survival rate, ranging between 12 to 15 months,. GBM manages to escape the immune system in a deadly symbiotic collaboration. Furthermore, it can also come from several cell types, not just glial cells. It is mainly present in adults aged 64 years and older, but can also occur in children, with a higher incidence in men compared to women. Gliomas can either be primary (precursor), or secondary (when a low-grade glioma is transformed) (30, 31). Studies have shown that patients with an isocitrate dehydrogenase (IDH) mutation have a longer survival and respond to chemotherapy and radiotherapy well unlike those who do not have the mutation (32).

Tumor-infiltrating immune cells are cells that have left the bloodstream to enter the tumor microenvironment. Their function may change throughout tumor progression, depending on the type of cells and their functional interactions. Indeed, immune cells may play a key role in tumor suppression or in tumor growth support, with specific effects on patient behavior (33).

### Tumor-Associated Macrophages (TAM)

They represent microglia which are intrinsic to the brain, and act by creating supporting stroma for the expansion and invasion of neoplastic cells. TAMs that are recruited into the tumor microenvironment of gliomas can release growth factors and cytokines in response to cancer cell activity (34). Thus, TAM infiltrates gliomas in moderate numbers and often exhibit an immunosuppressive phenotype and functional behavior (33).

### Natural Killer Cells (NK)

NK cells are the prototypes of innate lymphoid cells. They are characterized by large granular lymphocytes containing perforins and granzymes and have a destructive function. These cells are able to kill tumor cells using soluble molecules of the tumor necrosis factor (TNF) family (35). Additionally, NK cells have also been identified in primary and metastatic brain neoplasms, where they have a key role in suppressing brain tumors (36). The level of tumor infiltration by NK cells tends to remain low and their functionality often affected by factors released by tumors or other immunosuppressive cells (37, 38).

### Dendritic Cells (DC)

DC are professional antigen-presenting cells that are found upon recognition of the pathogen at the site of inflammation. In cancers, in addition to antigen presentation, mature dendritic cells (mDC) release cytokines and chemokines to induce tumor-specific T cell activation (39). CD11c+ DCs were studied extensively in GL261 mouse glioma model and showed little or no co-stimulatory molecules in addition to being unable to stimulate T cells. However, these cells favored the development of regulatory T cells (Treg). Analysis of the peripheral blood in glioma patients showed a decrease in numbers compared to healthy patients, suggesting that these cells may have been implicated in tumor pathogenesis (33).

**Figure 1:**
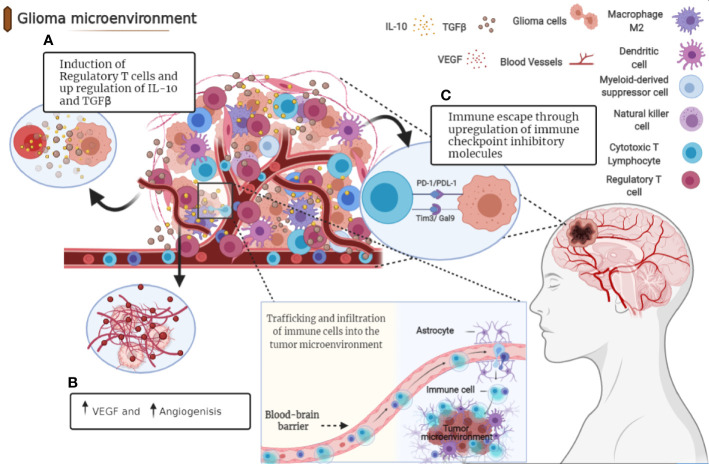
Immunosuppressive microenvironment of glioma. Tumor cells release molecules which contribute to multiple unique immunosuppression mediated by various cellular players in glioma microenvironment. **(A)** After recruitment to the tumor site, Tregs directly suppress the activity of cytolytic T cells and induce their apoptosis through secretion of various types of cytokines including IL-10 and TGFβ. **(B)** Angiogenesis is a pathologic hallmark of glioblastoma mainly mediated by vascular endothelial growth factor (VEGF). **(C)** Immune checkpoints suppress T cell function in glioma microenvironment through distinct mechanisms.

Usually, high levels of cytotoxic T cell-directed human glioma cells (CTL) are associated with increased antitumor activity, whereas high levels of helper T cells (particularly Th17) are thought to be associated with the role of promoting tumor development. Treg cells are a subset of CD4 T cells that express CD25 and FoxP3 (40). These play an important role in the regulation of the immune response by suppressing the proliferation of other T cells presented in the tumor microenvironment, through mechanisms directly dependent on cell contact or indirectly by the secretion of IL-10 and TGFβ (41, 42). In the tumor microenvironment, the production of specific chemokines and cytokines appears to be associated with preferential recruitment of Treg and subsequently poor prognosis (43) (**Figure 1**).

## Immune Checkpoint Inhibitors in Glioma Therapy

### Immune Checkpoints in Cancer

To escape immune surveillance, cancer cells have developed several mechanisms that induce a state of immune tolerance and evade immune destruction (44). One of the mechanisms is the use of the inhibitory and costimulatory receptors, called “immune checkpoints” (45). Clinical cancer treatment has become directed towards targeting T cell inhibitory receptors by using immune checkpoint inhibitors (ICI) (46, 47).

**Figure 2:**
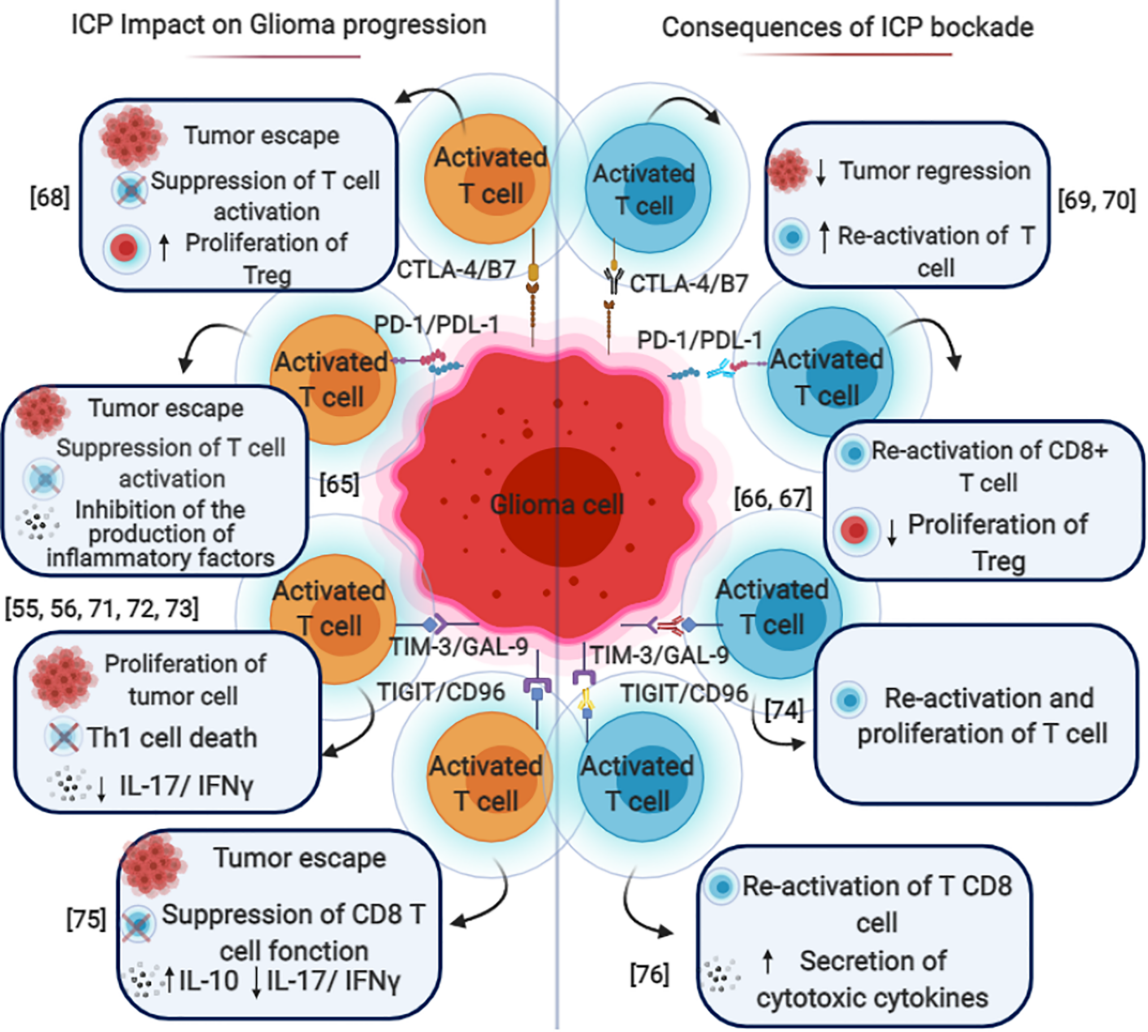
Immune checkpoint blockade in gliomas. The mechanisms by which various immune checkpoints promote each other and contribute to the immunosuppressive microenvironment in gliomas. PD-1/PD-L1, CTLA-4/B7, TIM3/GAL9, and TIGIT/CD96 expressed on different types of immune cells such as T cells (CD4 and CD8) Dendritic cells (DC), Natural killer cells (NK) B cells. These pathways could induce FoxP3 expression and promote tumor escape, cytotoxic cell inhibition and Treg conversion with the help of TGF-β and IL-10. The blockade of these immune checkpoint molecules through mono or combined therapy could be used as a potential therapeutic for glioma and especially glioblastoma.

Each tumor has a specific dynamic interaction of immune checkpoints, which highlights the importance of having a better understanding of the tumor-immune interactions in a hope to achieve and design a rational combination therapy specific to each tumor (48). Cancer immunotherapy differs from chemotherapy in that it aims to enhance the immune response in different stages of tumor progression and, in so doing, reducing patients’ clinical poor outcomes. Chemotherapy, on the other hand, destroys cancer cells directly (**Figure 2**) (49).

In recent years, inhibitors of CTLA-4 have shown remarkable success in cancer immunotherapy. Tremelimumab is a fully human monoclonal antibody to CTLA4 that has shown beneficial responses in clinical trials against different tumors, especially when combined with PD-1/PD-L1 blockade. However, in the case of glioblastoma, several studies reported that anti-CTLA-4 and/or anti-PD-1 antibodies exhibit no survival benefit compared to standard chemotherapy (50–53).

However, resistance to ICIs has become a common clinical phenotype that we currently do not have much knowledge about. Collaborative efforts are needed for a deeper understanding of biology to prevent, overcome or reverse this resistance (54).

The successful preclinical trials and the very positive results obtained with other tumors promoted the utilization of immune checkpoint inhibitors in GBM. Indeed, the survey of the NIH Clinical Trials Database (https://www.clinicaltrials.gov) performed on July 2018 showed registered trials of malignant glioma. (**Table 1**).

**Table 1:** Current clinical trials involving immune checkpoint blockade in human glioma.

Clinical trial	Title of the study	Study population	Phase	Intervention	Study design	Date
NCT01670890	Efficacy and Safety of TMZ Plus CDDP in the Patients With Recurrent Malignant Gliomas	Malignant Gliomas	Phase I	Drug: Temozolomide Drug: Temozolomide plus neoadjuvant CDDP	Allocation: Non-Randomized Intervention Model: Parallel Masking: None (Open Label). Primary Purpose: Treatment Assignment	August 2012
NCT03011671	Study of Acetazolamide With Temozolomide in Adults With Newly Diagnosed or Recurrent Malignant Glioma	Malignant Glioma of Brain	Phase I	Drug: Acetazolamide and Tolomozomide	Allocation: N/A Intervention Model: Single Group Assignment Masking: None (Open Label) Primary Purpose: Treatment	October 3, 2018
NCT03973879	Combination of PVSRIPO and Atezolizumab for Adults With Recurrent Malignant Glioma	Malignant Glioma	Phase I Phase II	Biological: PVSRIPO Drug: Atezolizumab	Allocation: N/A Intervention Model: Single Group Assignment Masking: None (Open Label) Primary Purpose: Treatment	February 2020
NCT00953121	Bevacizumab Plus Irinotecan Plus Carboplatin for Recurrent Malignant Glioma (MG)	Malignant Glioma	Phase II	Drug: bevacizumab and CPT-11 and Carboplatin	•Allocation: NonRandomized Intervention Model: Parallel Assignment Masking: None (Open Label) Primary Purpose: Treatment	September 2009
NCT02313272	Hypofractionated Stereotactic Irradiation (HFSRT) With Pembrolizumab and Bevacizumab for Recurrent High Grade Gliomas	Malignant Glioma	Phase I	Radiation: Hypofractionated Stereotactic Irradiation (HFSRT) Drug: Pembrolizumab Drug: Bevacizumab	Allocation: N/A Intervention Model: Single Group Assignment Masking: None (Open Label) Primary Purpose: Treatment	May 5, 2015
NCT02829931	Hypofractionated Stereotactic Irradiation With Nivolumab, Ipilimumab and Bevacizumab in Patients With Recurrent High Grade Gliomas	Malignant Glioma	Phase I	Radiation: Hypofractionated Stereotactic Irradiation Drug: Nivolumab Drug: Bevacizumab Drug: Ipilimumab	Allocation: N/A Intervention Model: Single Group Assignment Masking: None (Open Label) Primary Purpose: Treatment	August 22, 2016
NCT01891747	A Phase I Study of High-dose L-methylfolate in Combination With Temozolomide and Bevacizumab in Recurrent High Grade Glioma	Malignant Glioma	Phase I	Drug: Bevacizumab Drug: Temozolomide Dietary Supplement: Vitamin C	Allocation: N/A Intervention Model: Single Group Assignment Masking: None (Open Label) Primary Purpose: Treatment	July 2013
NCT00271609	Bevacizumab for Recurrent Malignant Glioma	Recurrent High-Grade Gliomas Malignant Gliomas	Phase II	Drug: Bevacizumab	Allocation: Randomized Intervention Model: Parallel Assignment Masking: None (Open Label) Primary Purpose: Treatment	December 2005
NCT02590263	Study Evaluating ABT-414 in Japanese Subjects With Malignant Glioma	Malignant Glioma Glioblastoma Multiforme	Phase I Phase II	Radiation: Whole Brain Radiation Drug: Temozolomide Drug: ABT-414	Allocation: NonRandomized Intervention Model: Single Group Assignment Masking: None (Open Label) Primary Purpose: Treatment	August 24, 2015
NCT00782756	Bevacizumab, Temozolomide and Hypofractionated Radiotherapy for Patients With Newly Diagnosed Malignant Glioma	Brain Cancer Malignant Glioma	Phase II	Other: radiotherapy (RT) in combination with temozolomide and bevacizumab	Allocation: N/A Intervention Model: Single Group Assignment Masking: None (Open Label) Primary Purpose: Treatment	October 28, 2008
NCT01738646	Ph II SAHA and Bevacizumab for Recurrent Malignant Glioma Patients	Recurrent Glioblastoma Multiforme Malignant Glioma Adult Brain Tumor	Phase II	Drug: Vorinostat Drug: Bevacizumab	Allocation: N/A Intervention Model: Single Group Assignment Masking: None (Open Label) Primary Purpose: Treatment	January 2013

Due to the recent COVID-19 outbreak, oncologists are wondering about the risk of administering ICIs to patients. The concern was mainly regarding the overlap between the possible pneumological toxicity from anti-PD-1/PD-L1 agents, which could be life threatening in the case of coronavirus-related interstitial pneumonia. The overall incidence rate of ICI-related pneumonitis ranges from 2.5–5% with anti-PD-1/PD-L1 monotherapy to 7–10% with anti-CTLA-4/anti-PD-1 combination therapy  (55).

### Immune Checkpoints in Glioma

Glioma cells secrete different types of chemokines, cytokines and growth factors that enhance infiltration of various cells such as astrocytes, pericytes, endothelial cells, circulating progenitor cells, and a range of immune cells including microglia, peripheral macrophages, myeloid-derived suppressor cells (MDSC), CD4+ T cells as well as Treg cells into the tumor (56–59). However, identification of these factors may facilitate the improvement of glioma immunotherapy as immunomodulatory and immune evasion mechanisms used by glioma cells.

Glioma cells express the ligands which recognize and bind to partner proteins (receptor) on the surface of immune cells. Subsequent preclinical research showed their important role in the maintenance of peripheral immune tolerance and control overreaction to inflammatory responses. In fact, in glioma case different immune checkpoint molecules have been described such as CTLA-4, PD-1, TIM-3, and LAG-3; each of these receptors has corresponding ligands (60).

### PD-1/PD-L1

**Figure 3:**
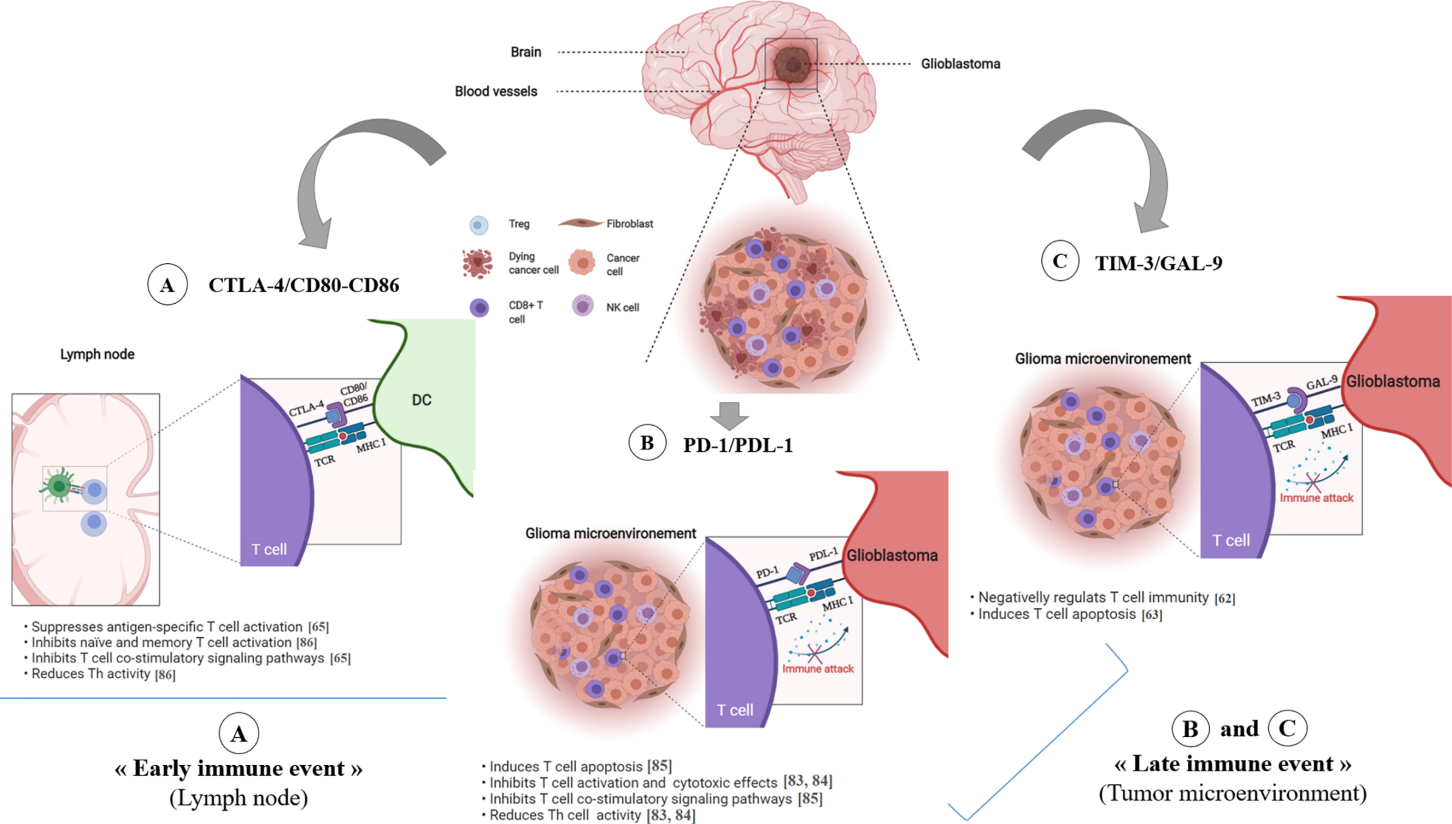
Immune checkpoint inhibitors in glioblastoma. The CTLA-4 immune checkpoint **(A)** operates early during the priming phase of the immune response. CTLA-4 preferentially binds to CD80/CD86 on the surface of APCs, thus leading to decreased T-cell activation and proliferation in the context of tumor antigen presentation. The T cell-expressed inhibitory PD-1 receptor interacts with PD-L1 **(B)**, which is expressed on tumor cells. Engagement of PD-1 and PDL-1, in the context of tumor antigen- presentation by MHC class I molecules, induces T cell apoptosis, inhibits T cell activation/cytotoxicity, promotes Tregs proliferation and blocks the production of inflammatory mediators, resulting in T cell inactivity. TIM-3/GAL-9 pathway **(C)** negatively regulates T cell immunity and induces T cell apoptosis. *ICP, Immune Checkpoint.

In 2014, the FDA approved immune checkpoint PD-1 targeting. The anti-PD-1 and anti-PD-L1 mAbs act to block distinct inhibitory signals that unleash T cells to have the ability to eradicate tumors (46, 61–63). In the case of GBM, PD-1 is expressed on T cells, B cells, tumor associated macrophages (TAMs), MDSCs, and NK cells (64). Immunotherapy is used to target the PD-1/PD-L1 pathway (**Figure 3B**) to trigger an antitumor immune response (65–67). The immunosuppressive tumors can then be resected, followed by a continuation of immunotherapy to enhance the functions of the TILs (64, 68).

The BBB is a factor that requires attention, unfortunately numerous drugs that have been tested in clinical trials for GBM patients have failed due to the lack of successful drug delivery across the BBB (69), thus affecting their therapeutic efficacy on intracranial tumors. However, despite recent studies showing that anti-PD-1 antibodies cannot cross the BBB, it is in fact not entirely true. Anti-PD-1 have a mechanism which enables them to bind irreversibly to PD1 or CTLA-4 on peripheral lymphocytes and ultimately penetrate the BBB. Once they have passed the BBB, they can bind TILs which occupy the intracranial tumors (70).

In a previous study, GBM tumor-bearing mice were treated with anti-PD-1 antibody or with a combination of anti-PD-1 and anti-CTLA-4 antibodies. Significant improvement in survival was noted in WT and CD73−/− mice treated with a combination of anti-PD-1 compared to controls (48). Furthermore, GBM patients who received anti-PD-1 therapy showed a persistence of immunosuppressive CD73 high myeloid subsets. Benefits of therapy by immune checkpoint inhibitors in a CD73−/− mouse model should be explored further (48).

### TIM3/GAL9

T cell immunoglobulin and mucin domain-containing molecule 3 (Tim-3) is an inhibitory receptor expressed on the surface of T cells and plays a key role in the inhibition of T cell responses against tumors (71) (**Figure 3C**). Galectin-9 has been identified as a ligand for Tim-3, and upon binding, it results in the apoptosis of T cells and a negative regulation of T cell immunity (72–75).

A recent study investigated the expression of Tim-3 and galectin-9 in glioma tissues and showed that there is an association between the expression of these immune checkpoint receptors and the malignancy of gliomas (50). The expression level of Tim-3 on healthy PBMCs was low and the expression of galectin-9 on non-cancerous brain tissues also followed a similar pattern. However, Tim-3 and galectin-9 were highly expressed in TILs and glioma tissues (50).

Yuan et al. demonstrated that Gal9 was highly expressed in the core than in the periphery of tumors in GBM patients, and that those with high expression of Gal9 had significantly shorter survival than those with low expression (76). This suggests that Gal9 is closely related to glioma patient’s prognosis and plays a key role in the malignant progression of GBM (76, 77).

### CTLA4

In 2011, the FDA approved an immune checkpoint agent, ipilimumab, a monoclonal antibody (mAbs) that targeted the checkpoint molecule CTLA4. This was based on a randomized phase III clinical trial that demonstrated an improved survival rate with durable clinical response for patients with advanced melanoma (46). As shown in **Figure 3A**; CTLA-4 suppresses antigen-specific T-cell activation and is expressed on activated T cells and CD4^+^Foxp3^+^ Tregs (78, 79).

A higher expression of CTLA-4 was observed in more severe grades of glioma, and this indicates that it is linked to a worse prognosis (80, 81). It was also found that CTLA-4 significantly correlates with PD-1, CD40, and ICOS (53). Besides, it was tightly associated with CXCL12, CXCR3, CXCR6, and TIGIT (a new promising immune checkpoint-related protein). The combination of these molecules can potentially enhance the efficacy of CTLA-4 blockade in cancer immunotherapy (53). Furthermore, it has been observed that the CTLA-4 antibodies do not cross the BBB. To solve that, Galstyan et al. attempted to combine nanotechnology and immunotherapy. They delivered nanoscale immunoconjugate (NIC) drugs across the BBB to treat GBM (82). They used a versatile drug carrier and poly (β-L-malic acid) (PMLA),a natural polymer obtained from the slime mold *Physarum polycephalum*, to deliver covalently conjugated CTLA-4 and PD-1 antibodies to brain tumor cells. This resulted in a local immune system activation and a prolonged survival of intracranial GBM in GL261-bearing mice (82). Currently, clinical trials of anti-CTLA-4 (ipilimumab) and anti-PD-1 (nivolumab) are being performed in patients with glioma, testing the safety, toxicities, and efficacy (78).

### LAG3

Lymphocyte activation gene‐3 (LAG3), also known as CD223, is a potential cancer immunotherapeutic target because of its negative regulatory role on T cells (83). It is expressed on activated human T and NK cells, and is an activation marker for CD4^+^ and CD8^+^ T cells (83). Mair et al. have shown that LAG-3^+^ TILs are rarely observed in IDH-wt and absent in IDH-mt glioma (84). However, these cells are more present in an active inflammatory microenvironment but according to LAG-3^+^ TIL infiltration; there was no difference in overall survival (84).

### TIGIT/CD96

T-cell immunoglobulin and ITIM domain (TIGIT) and CD96 are co-inhibitory receptors. TIGIT is expressed on conventional αβ T cells upon activation, memory T cells, regulatory T cells (Treg), both follicular helper T cells (TFH) and follicular regulatory T cells (TFR), NKT and NK cells (85). However the expression of CD96 has been reported primarily on conventional αβ and γδ T cells, NK cells, and NKT cells (85). Hung et al. have found high levels of TIGIT expression on CD8^+^ and CD4^+^ TILs in glioma patients. They have also shown that anti-TIGIT therapy alone had no significant effect on the survival rate in the GBM mouse model (86). However, combination therapy using anti-TIGIT and anti-PD-1 showed a significant increase in survival (87, 88); this was carried out through modulation of both the T cell and myeloid compartments (86). Additionally, elevated frequencies of CD8 + and CD4 + T cells with double expression of IFNγ and TNFα have also been reported during combination therapy, compared to monotherapy and control groups (86).

Zhang et al. showed that high expression of *CD96* was present in the malignant molecule phenotype, including IDH wild type and mesenchymal subtype. They also stressed that it had a positive association with inflammatory activities (89). Indeed, *CD96* showed a high concordance with immune checkpoints such as *PD-1*, *CTLA-4*, *TIGIT*, *TIM-3*, *NR2F6*, and *GITR*, which would suggest a potential synergism (89). In addition to that, they discovered that higher *CD96* expression predicted worse survival rates in glioma and GBM patients overall. This implied that *CD96* blockade may significantly improve the prognosis of glioma patients (89) (**Figure 2**).

## Conclusion

In the present review, we managed to collect further evidence which demonstrates that the immune system is involved in glioma physiopathology and describes the general concept of immune response within the glioma microenvironment.

The immune cells are highly inhibited in the glioma microenvironment through various mechanisms, including immune checkpoint inhibitors. Undoubtedly, the discovery of immune checkpoints such as CTLA-4 and PD-1 played a key role in the development of cancer immunotherapy. Although these molecules were originally discovered as molecules with a role in the activation and apoptosis of T cells, subsequent preclinical studies showed their important role in the maintenance of peripheral immune tolerance. In addition, several predictive glioma biomarker studies are completed and many are underway. Indeed, the clinical validation of the identified biomarkers is necessary. Lastly, investigations in glioma immunotherapy should decipher adequate ways to facilitate BBB crossing of these therapeutic molecules in order to potentially benefit from current and future therapies. Integrated approaches should also be developed to identify patient-specific choices for checkpoint monotherapies or combination therapies.

## Author Contributions

AG, SK, MT, KR, and SR wrote the review manuscript sections. AB wrote the review manuscript and supervised the study. All authors contributed to the article and approved the submitted version.

## Funding

This work was supported by The Moroccan Ministry of Higher Education and Research (grant PPR1, type B and grant Al khawarizmi for AB).

## Conflict of Interest

The authors declare that the research was conducted in the absence of any commercial or financial relationships that could be construed as a potential conflict of interest.
